# Supplementary material for: The genome sequence of Veronica verna L., 1753 (Lamiales: Plantaginaceae)
Source: Wellcome Open Res. 2026 Jun 27;11:395. [Version 1] doi: 10.12688/wellcomeopenres.26894.1 (PMC13562990; doi:10.12688/wellcomeopenres.26894.1)
Supplement: Peer review history

# Reviewer report: Reviewer response for version 1

**DOI:** 10.21956/wellcomeopenres.29613.r165466

**Published:** 2026-08-31

**Authors:** Zhen Li, Haiyun Fan

**Affiliations:** 1 Plant Biotechnology and Bioinformatics, Ghent University (Ringgold ID: 26656), Ghent, Flanders, Belgium; 2 Ghent University, Ghent, Belgium

This paper presents a high-quality genome assembly of *Veronica verna* (Plantaginaceae), with robust chromosomal scaffolding and comprehensive organelle genome assemblies. The assembly quality metrics are impressive, with thorough evaluations including flow cytometry, GenomeScope analysis, and Hi-C confirmation. Gene annotation is complete, identifying a substantial set of protein-coding and non-coding genes. All data are publicly accessible in international repositories with clear accession numbers. Overall, this genome assembly represents a valuable resource for Plantaginaceae, and the paper should be indexed.

 Minor comments:

1. The phrase ‘with deeply lobed upper leaves’ is imprecise and could be confused with the upper bracts, which are typically narrow and undivided. The characteristic of being deeply lobed applies to the middle to upper stem leaves. Please revise the description for accuracy, for example, to ‘with deeply lobed middle stem leaves’.
2. Specimen consistency for genome and Hi-C sequencing: Two different specimens were used (KDTOL10750 for genome, KDTOL10751 for Hi-C), but it is unclear whether both originated from the same cultivated source. I recommend clarifying this, as a brief statement confirming they are from the same batch would suffice.
3. Genome size estimate inconsistency: The text reports a GenomeScope-estimated haploid genome size of 550.69 Mb, while Figure 2 displays 550,114,855 bp (equivalent to 550.11 Mb). Please clarify the source of this discrepancy and correct accordingly.

Are sufficient details of methods and materials provided to allow replication by others?

Yes

Is the rationale for creating the dataset(s) clearly described?

Yes

Are the datasets clearly presented in a useable and accessible format?

Yes

Are the protocols appropriate and is the work technically sound?

Yes

Reviewer Expertise:

evolutionary biology; bioinformatics; genomics

We confirm that we have read this submission and believe that we have an appropriate level of expertise to confirm that it is of an acceptable scientific standard.

# Reviewer report: Reviewer response for version 1

**DOI:** 10.21956/wellcomeopenres.29613.r159750

**Published:** 2026-07-10

**Authors:** Peng-Fei Ma

**Affiliations:** 1 Chinese Academy of Sciences, Kunming, China

In this study, a species from the Plantaginaceae family, *Veronica verna, *was sequenced and assembled. The assembly is high quality with 98.85% of sequences scaffolded into 8 chromosomes. The assembly statistics of total number of scaffolds and N50 looks fine and quality metrics are also very high. Finally, a total of 22,903 protein-coding genes were annotated.

Are sufficient details of methods and materials provided to allow replication by others?

Yes

Is the rationale for creating the dataset(s) clearly described?

Yes

Are the datasets clearly presented in a useable and accessible format?

Yes

Are the protocols appropriate and is the work technically sound?

Yes

Reviewer Expertise:

Plant genomics and evolution

I confirm that I have read this submission and believe that I have an appropriate level of expertise to confirm that it is of an acceptable scientific standard.
